# High resolution 3D laboratory x-ray tomography data of femora from young, 1–14 day old C57BL/6 mice

**DOI:** 10.1016/j.dib.2015.04.001

**Published:** 2015-04-20

**Authors:** Emely L. Bortel, Georg N. Duda, Stefan Mundlos, Bettina M. Willie, Peter Fratzl, Paul Zaslansky

**Affiliations:** a Max Planck Institute of Colloids and Interfaces, Department of Biomaterials, Research Campus Golm, Am Mühlenberg 1, 14476 Potsdam, Germany; b Berlin-Brandenburg School for Regenerative Therapies (BSRT), Charité-Universitätsmedizin Berlin, Campus Virchow Klinikum, Augustenburger Platz 1, 13353 Berlin, Germany; c Julius Wolff Institute, Charité-Universitätsmedizin Berlin, Campus Virchow Klinikum, Augustenburger Platz 1, 13353 Berlin, Germany; d Max Planck Institute for Molecular Genetics, Berlin, Ihnestraße 63-73, 14195 Berlin, Germany; e Institute of Medical and Human Genetics, Charité-Universitätsmedizin Berlin, Campus Virchow Klinikum, Augustenburger Platz 1, 13353 Berlin, Germany

**Keywords:** Mouse bone, x-ray tomography

## Abstract

This data article contains high resolution (1.2 µm effective pixel size) lab-based micro-computed tomography (µCT) reconstructed volume data of the femoral mid-shafts from young C57BL/6 mice. This data formed the basis for the analyses of bone structural development in healthy mice, including closed and open porosity as reported in Bortel et al. [1]. The data reveals changes seen in bone material and porosity distribution observed when mouse bones transform from porous scaffolds into solid structures during normal organogenesis.

## Specifications table

**Table:** 

Subject area	*Biology*
More specific subject area	*Structural biology*
Type of data	*3D x-ray images*
How data was acquired	*3D micro-computed-tomography (Skyscan 1172, Bruker, Kontich, Belgium)*
Data format	*reconstructed*
Experimental factors	*Mice bones were dissected, the soft tissue partially removed and stored in an ethanol environment (sealed PMMA vial).*
Experimental features	*Samples were scanned with a source voltage of 60* *kV, 160 µA, no filter and an effective resolution of 10* *µm (600 projections) and 1.2 µm (3600 projections).*
Data source location	*Germany*
Data accessibility	*Data in Brief Dataverse*10.7910/DVN/29628

## Value of the data

• The data contain high resolution 3D density distributions of the microstructures of the forming mineralized femur bones in growing mice
• The data provide a reference for an important part of the skeleton of normal C57BL/6 mice and may serve to benchmark disease or treatment models by other groups.
• In addition to understanding the 3D mineral distribution changes with age, important dynamics in porosity evolution during normal mouse bone growth is observed. Porosity arises due to voids typically containing blood vessels or osteocyte lacunae.

## 1 Data, experimental design, materials and methods

C57BL/6 mice (sacrificed after 1, 3, 7, 10 and 14 days post-natal, *n*=3/age) were obtained from a colony at the Max Planck Institute for Molecular Genetics (Berlin, Germany) and left femora were dissected. The soft tissue was partially removed and the samples were stored in sealed PMMA vials containing an ethanol environment at 4 °C. Samples were scanned with a polychromatic x-ray source lab-based micro-computed-tomograph (Skyscan 1172, Bruker, Kontich, Belgium). To obtain a high contrast, a source voltage of 60 kV and current of 160 µA was applied (without any filter) for the femora from 1, 3, 7, and 10 day old mice. To overcome the higher absorption in the 14 day old mice femora, due to their dense structure, a source voltage of 75 kV and a current of 133 µA were used. The high resolution scans made available (Harvard Dataverse 10.7910/DVN/29628, [2]) were produced using 3600 radiographs with 1.2 µm effective pixel size. Medium resolution scans (10 µm, 900 projections) were used to locate the high resolution scans. Reconstruction was performed using a standard back-projection Feldkamp algorithm [3]. The manufacturer software was used (NRecon 1.6.8, Bruker, Kontich) and beam hardening and ring artefact correction factors were adapted according to the observed severity of artefacts in each scan and spanned 10–20% and 20–100%. A mild 3×3 pixel Gaussian filter was applied to reduce noise. All samples were aligned along the bone long-axis.
